# 5-HT_7_ receptor-dependent intestinal neurite outgrowth contributes to visceral hypersensitivity in irritable bowel syndrome

**DOI:** 10.1038/s41374-022-00800-z

**Published:** 2022-05-18

**Authors:** Wen-Ying Chang, Yi-Ting Yang, Meng-Ping She, Chia-Hung Tu, Tsung-Chun Lee, Ming-Shiang Wu, Chin-Hung Sun, Ling-Wei Hsin, Linda Chia-Hui Yu

**Affiliations:** 1 Graduate Institute of Physiology, National Taiwan University College of Medicine, Taipei, Taiwan ROC; 2 Department of Internal Medicine, National Taiwan University Hospital and College of Medicine, Taipei, Taiwan ROC; 3 Department of Tropical Medicine and Parasitology, National Taiwan University College of Medicine, Taipei, Taiwan ROC; 4 Graduate Institute of Pharmacy, National Taiwan University School of Pharmacy, Taipei, Taiwan ROC; 5 Center for Innovative Therapeutics Discovery, National Taiwan University, Taipei, Taiwan ROC

**Keywords:** Cellular neuroscience, Target validation

## Abstract

Irritable bowel syndrome (IBS) is characterized by visceral hypersensitivity (VH) associated with abnormal serotonin/5-hydroxytryptamine (5-HT) metabolism and neurotrophin-dependent mucosal neurite outgrowth. The underlying mechanisms of VH remain poorly understood. We investigated the role of 5-HT_7_ receptor in mucosal innervation and intestinal hyperalgesia. A high density of mucosal nerve fibres stained for 5-HT_7_ was observed in colonoscopic biopsy specimens from IBS patients compared with those from healthy controls. Staining of 5-HT_3_ and 5-HT_4_ receptors was observed mainly in colonic epithelia with comparable levels between IBS and controls. Visceromotor responses to colorectal distension were evaluated in two mouse models, one postinfectious with *Giardia* and subjected to water avoidance stress (GW) and the other postinflammatory with trinitrobenzene sulfonic acid-induced colitis (PT). Increased VH was associated with higher mucosal density of 5-HT_7_-expressing nerve fibres and elevated neurotrophin and neurotrophin receptor levels in the GW and PT mice. The increased VH was inhibited by intraperitoneal injection of SB-269970 (a selective 5-HT_7_ antagonist). Peroral multiple doses of CYY1005 (a novel 5-HT_7_ ligand) decreased VH and reduced mucosal density of 5-HT_7_-expressing nerve fibres in mouse colon. Human neuroblastoma SH-SY5Y cells incubated with bacteria-free mouse colonic supernatant, 5-HT, nerve growth factor, or brain-derived neurotrophic factor exhibited nerve fibre elongation, which was inhibited by 5-HT_7_ antagonists. Gene silencing of *HTR7* also reduced the nerve fibre length. Activation of 5-HT_7_ upregulated *NGF* and *BDNF* gene expression, while stimulation with neurotrophins increased the levels of tryptophan hydroxylase 2 and 5-HT_7_ in neurons. A positive-feedback loop was observed between serotonin and neurotrophin pathways via 5-HT_7_ activation to aggravate fibre elongation, whereby 5-HT_3_ and 5-HT_4_ had no roles. In conclusion, 5-HT_7_-dependent mucosal neurite outgrowth contributed to VH. A novel 5-HT_7_ antagonist could be used as peroral analgesics for IBS-related pain.

## Abstract (web summary)

Current treatments, which mostly aim to restore bowel habits, are ineffective for intestinal pain in irritable bowel syndrome (IBS). Abnormal gut-derived serotonin metabolism and mucosal neurite outgrowth are linked to visceral hypersensitivity. Enhanced mucosal innervation dependent on 5-HT_7_ contributes to hyperalgesia in two IBS-like mouse models. A positive-feedback loop between serotonin and neurotrophin is involved in intensifying nerve fiber elongation. A novel 5-HT_7_ receptor antagonist may be administered orally as an intestinal analgesic for IBS-related pain.

## Introduction

Irritable bowel syndrome (IBS) is mainly characterized by recurrent abdominal pain associated with bowel habit changes in the absence of identifiable organic causes and macroscopic lesions^1^. Diverse risk factors, including psychological stress, intestinal infection, and inflammation history, are associated with the development of IBS symptoms^2,3^. Although the defecation pattern may vary from diarrhoea to constipation, a lower pain threshold to intestinal distension, referred to as visceral hypersensitivity (VH), is reported in all IBS patients^4,5^. Current medical treatments are mostly aimed at restoring bowel habits but are ineffective for intestinal pain. The underlying mechanisms of intestinal hyperalgesia in IBS remain poorly understood.

Altered metabolism of gut-derived serotonin/5-hydroxytryptamine (5-HT) was one of the first biomarkers that helped classify IBS as an intestinal disorder instead of a mental illness^6–8^. The monoamine neurotransmitter 5-HT, originally identified in the brain, is now recognized to be primarily (~90%) secreted by enteric neurons and enteroendocrine cells to control bowel movement and pain sensation under physiological conditions^9^. Experimental models showed that intracolonic and intraperitoneal administration of 5-HT caused intestinal hyperalgesia^10–12^. Nuclei of the enteric neurons are normally localized in the submucosal and myenteric plexuses, and few nerve fibres are observed in the mucosal region. Nevertheless, recent evidence has indicated that increased nerve fibre density and elevated levels of neurotrophins such as nerve growth factor (NGF) and brain-derived neurotrophic factor (BDNF) were observed in mucosal biopsy specimens which correlated with abdominal pain scores in IBS patients^13–16^. Previous work from our laboratory and other groups has demonstrated enhanced innervation of the gut mucosa and increased afferent sensitivity in mouse models of postinfectious and postinflammatory pain^17,18^. Therefore, we speculated that 5-HT, traditionally regarded as a neurotransmitter, may also be involved in the gut neuroplasticity of IBS patients.

Among the seven serotonin receptor subtypes, increased transcript levels of 5-HT_3_ and 5-HT_7_ have been documented in colorectal tissues of diarrhoea-predominant IBS patients and rodent models, albeit inconsistent results were noted among the references^19–21^. Drugs targeting 5-HT_3_ and 5-HT_4_ have been used for treatment of IBS symptoms but are associated with ischaemic side effects^22,23^. 5-HT_7_ is a G-protein coupled receptor identified in enteric neurons, smooth muscles, and dendritic cells in the colon^24–26^. Stimulation of 5-HT_7_ resulted in exaggerated relaxation of intestinal circular smooth muscle and activation of excitatory postsynaptic potential in colonic myenteric afferents^27,28^. Whether 5-HT_7_ receptor plays a role in VH and through which mechanisms remain unclear.

In the current study, mucosal nerve fibre density and expression patterns of 5-HT receptor subtypes were examined in colonoscopic biopsy specimens of IBS patients. The role of 5-HT_7_ in VH and the mechanisms linking serotonin and neurotrophin pathways for intensifying mucosal neurite outgrowth were investigated using two mouse models with IBS-like pain. Moreover, a novel 5-HT_7_ receptor ligand administered perorally was evaluated for its effects on IBS-related pain.

## Materials and methods

### Human colonoscopic specimens

Mucosal biopsy specimens were collected from healthy control (HC) subjects (*n* = 12) and diarrhoea-predominant IBS patients (*n* = 13) by colonoscopic procedures at National Taiwan University Hospital (NTUH) (Supplementary Table 1). Eligibility of IBS patients was evaluated based on clinical symptoms that fulfilled the Rome IV criteria, while HC subjects should be free of diarrhea, constipation, or abdominal symptoms for at least three months^29^. For both HC and IBS groups, four specimens were obtained from ascending colon and another four from descending colon. Subjects with a history of bowel surgery or cholecystectomy, elevated white blood cell count, serum C-reactive protein, erythrocyte sedimentation rate, or whose colonoscopy revealed infectious or inflammatory lesions were not included. Written informed consent was obtained from all study subjects, and approval for this study was granted by the Research Ethics Committee of NTUH (201611075RINA). Cryofixed specimens were immunostained for protein gene product 9.5 (PGP9.5) and 5-HT receptor subtypes, and then the cell nuclei were counterstained with a Hoechst dye (see below). Negative controls including isotype antibody or those omitting the primary antibody were performed to confirm specific staining. Immunofluorescence images were captured under a Zeiss microscope equipped with a camera. Staining intensity of PGP9.5 in two to three images per individual and a total of 30–32 images per group was scored by a blinded observer according to the following criteria: 0, negative staining in the lamina propria; 1, <10% of the crypts showed positive staining in the lamina propria as sparsely distributed puncta; 2, 11–20% was positive as sparsely distributed puncta; 3 to 5, 21–50% was positive with some densely distributed puncta; 6 to 8, 51–80% was positive as densely distributed puncta and fibre-like staining; 9 to 10, >81% was positive as densely distributed fibre-like staining.

### Animals

Specific pathogen-free C57BL/6 male mice (4–6 weeks of age) were obtained from the Animal Centre of the NTU College of Medicine. The animals were housed in a temperature-controlled room (20 ± 2 °C) on a 12/12-h light/dark cycle and provided regular chow and water ad libitum. All experimental procedures were approved by the Institute of Animal Care and Use Committee.

### Reagents

SB-269970 hydrochloride (SB7, a selective 5-HT_7_ antagonist) and CYY1005 (CYY, a novel 5-HT_7_ ligand) were intraperitoneally (i.p.) or perorally (p.o.) administered to mice at the indicated doses for analysis of intestinal pain or added to neuronal cells for analysis of nerve fibre length. In addition, alosetron (ALN, a selective 5-HT_3_ antagonist) and GR125487 (GR, a selective 5-HT_4_ antagonist) were used. SB7 and ALN were purchased from Sigma-Aldrich (St. Louis, MO, USA) and GR was purchased from Tocris Bioscience (Minneapolis, MN, USA). CYY1005 (PCT# WO2018157233 (A1)) was chemically synthesized by the laboratory of Professor Hsin LW, School of Pharmacy, NTU.

### Mouse models

Two mouse models of IBS-like VH were used in the study, including one generated by *Giardia* postinfection combined with psychological stress^17,30–32^ and the other generated by postinflammation from intracolonic injection of the colitogenic agent 2,4,6-trinitrobenzene sulfonic acid (TNBS)^18,33^.

The first model (designated the GW model) was generated by subjecting mice to water avoidance stress (WAS) in the post-clearance phase of giardiasis (G)^17,30,31^. The use of multiple triggers is to mimic the heterogeneous risk factors in patients^17,34^. Uninfected unstressed controls (Ctrl) were pair-fed saline and left in cages unhandled. Briefly, the mice were orally gavaged with 10^7^ *Giardia lamblia* trophozoites (strain GS/M) suspended in 0.2 ml of sterile phosphate-buffered saline. Clearance of *Giardia* trophozoites in the intestine three weeks after inoculation was observed in our pilot study, confirming the self-limiting status of infection. During the sixth week postinfection, at which point trophozoites could not be detected in the intestine, mice were subjected to WAS for 1 hr/day for ten consecutive days. The perception of psychological stress was confirmed by measuring the blood corticosterone levels of the animals using an ELISA kit (Cayman Chemical, Ann Arbor, MI, USA). On the last day of stress exposure, the intestinal pain of the mice was measured. To test analgesic effects in the GW model, mice were administered a single dose of chemical reagents i.p. or p.o. at a concentration of 5 mg/kg (unless otherwise stated) 90 min prior to intestinal pain measurement. For other experiments, the reagents were administered multiple times at a concentration of 0.5, 1, or 3 mg/kg 30 min before the start of each stress session for 10 consecutive days, and intestinal pain was measured immediately after the last stress session.

In the second model, postinflammatory pain was measured after the resolution of colitis, which was induced by intracolonic injection of 10 mg/mL TNBS (Sigma-Aldrich) dissolved in 40% ethanol in 0.2 ml of saline (designated the post-TNBS (PT) model)^18,33^. Sham controls (Sham) were intracolonically injected with the same volume of saline. Intestinal pain levels and inflammatory parameters (i.e., myeloperoxidase activity and histopathological scores) were measured on various days in our pilot study, and 24 days PT was chosen as the time point to measure persistent pain in the absence of inflammation. To test the analgesic effects of reagents in the PT model, the mice were i.p. or p.o. administered a single dose of chemical reagents at a concentration of 5 mg/kg (unless otherwise stated) 90 min before or multiple doses (3 mg/kg) for 10 consecutive days prior to pain measurement.

### Assessment of intestinal pain sensation

Abdominal pain was assessed by measuring the visceromotor responses (VMRs) of mice to colorectal distension (CRD) following a previously described protocol^17,35^. Briefly, electrodes were implanted in the abdominal external oblique muscles and exteriorized onto the back of the necks of mice at least 15 days prior to VMR measurement. Conscious mice were habituated to a plexiglass cylinder for 30 min per day for 3 consecutive days before VMR measurement to accustom the animals to partial constraint. For recording, the electrodes were connected to an electromyogram (EMG) acquisition system (AD Instruments). The colon was distended by inflating a balloon catheter inserted intra-anally such that it ended 1.5 cm proximal to the anus. The mice were subjected to four 10-second distensions (15, 40, and 65 mmHg) at 3-minute intervals. EMG activity was amplified and digitized using a transducer connected to a P511 AC amplifier (Grass Instruments) and a Powerlab device with Chart 5 software (AD Instruments). EMG activity was rectified, and responses were recorded as the increase in the area under curve (AUC) of the EMG amplitude during CRD versus the baseline period.

### Histopathological examination

Intestinal tissues were fixed in 4% paraformaldehyde (PFA) and embedded in paraffin wax with proper orientation of the crypt to villus axis before sectioning. Sections of 5-μm thickness were deparaffinized with xylene and graded ethanol, stained with hematoxylin and eosin (H&E). The sections were observed under a light microscope and the histopathological scores were determined in a blinded fashion^36–38^.

### Intestinal myeloperoxidase activity

Intestinal segments were suspended in 0.5% hexadecyltrimethylammonium buffer in 50 mM potassium phosphate buffer (PPB, pH = 6) at a ratio of one gram of tissue to 10 ml of buffer, and then homogenized and sonicated on ice. The lysate was centrifuged at 12,000 × *g* for 20 min. The supernatant (7 µl volume) was diluted with 200 µl of reactive buffer (PPB solution containing 0.167 mg/ml of *o*-dianisidine dihydrochloride and 0.0005% of H_2_O_2_ (Sigma)) in 96-well plates. The enzyme concentration was determined by the absorbance at 460 nm measured every 50 s over a 5-minute period. One unit of myeloperoxidase (MPO) activity was defined as the quantity of enzyme degrading 1 µmole of H_2_O_2_/min at 25 °C, and was expressed as Unit/mg of tissue.

### Immunofluorescence staining

Cryofixed sections of human biopsies and mouse colonic tissues were washed in saline twice, and quenched with 50 mM NH_4_Cl (Sigma) for 10 min. The tissues were incubated with 0.1% Triton X-100 (Sigma) for 30 min and blocked in fetal bovine serum (FBS) (Biological Industries, Kibbutz Beit-Haemek, Israel) for 1.5 h. The tissues were then stained with rabbit polyclonal anti-PGP9.5 (#GTX109637) (1:400, GeneTex, Irvine, CA, USA), anti-5-HT_7_ (#ab61562) (1:200, Abcam, Burlingame, CA, USA), anti-5-HT_3_ (#ab13897) (1:100, Abcam), and anti-5-HT_4_ (#ab60359) (1:200, Abcam) as primary antibodies or with isotype control antibodies overnight at 4 °C, followed by secondary antibodies anti-rabbit IgG conjugated to Alexa488 or Alexa594 (1:1000; Cell Signaling, Beverly, MA, USA) for 1 h at room temperature. After washing, the sections were counterstained with a Hoechst dye (1 μg/ml in PBS, Sigma) for 30 min in the dark to show cell nuclei for tissue orientation. The images were captured under a Zeiss microscope for quantification of fluorescence intensity by using an imaging software (Axio Vision SE64, Zeiss, Oberkochen, Germany). Fluorescence intensity per area was quantified in 5 images of the colonic mucosa per mouse for five mice per group. A total of 25 images from each mouse group were used for comparison.

Human cell lines grown on coverslips were fixed with 4% PFA for 30 min, permeabilized with 0.5% Triton X-100 for 5 min and quenched with 50 mM NH_4_Cl for 10 min. After blocking with 1% BSA for 1 h, cells were stained with primary antibodies including anti-PGP9.5 (#ab72911) (1:1000, Abcam), anti-5-HT_7_ (#ab137493) (1:250, Abcam), and anti-tryptophan hydroxylase 2 (TPH2) (#ab111828) (1:500, Abcam). The cells were then incubated with fluorescence-conjugated secondary antibodies, followed by a Hoechst dye. The images were captured for quantification of fluorescence intensity under a Zeiss microscope.

### Western blotting

Proteins were extracted from the intestinal mucosa or cultured cells with complete radioimmunoprecipitation assay buffer and subjected to sodium dodecyl sulfate/polyacrylamide gel electrophoresis (4–13% polyacrylamide)^39,40^. The resolved proteins were then electrotransferred onto polyvinylidene fluoride membranes with a semi-dry blotter. The blots were blocked with 5% (w/v) nonfat dry milk in Tris-buffered saline (TBS) with Tween 20 (TBS-T; 0.1% (v/v) Tween-20 in TBS, Sigma) for 1 h, washed with TBS-T, and incubated with a primary antibody at 4 °C overnight. The membranes were washed and incubated with a secondary antibody for 1 h. After washing, the membranes were incubated with chemiluminescent solution, and signals were detected. The primary antibodies used included rabbit polyclonal anti-5-HT_7_ (#ab13898) (1:500, Abcam) and mouse monoclonal anti-β-actin (1:5000, Sigma). The secondary antibodies used were horseradish peroxidase-conjugated anti-rabbit or mouse IgG (1:1000, Cell Signaling).

### Polymerase chain reaction

Quantitative polymerase chain reaction (qPCR) was performed using an Applied Biosystems StepOnePlus Real-Time PCR System (Applied Biosystems, Waltham, MA, USA). Total RNA (2 μg) was extracted from tissues and cell samples, and was reverse transcribed with oligo(dT)_15_ using RevertAid™ First Strant cDNA Synthesis kit (ThermoFisher, Waltham, MA, USA) in 20 μL reaction volume. The PCR reaction mixture consisted of 50 ng of RT product, 10 μL of Power SYBR Green PCR Master Mix and 125 nM specific primer pairs in a final reaction volume of 20 μL. The primer pairs for mouse and human cells were designed in this study based on the NCBI nucleotide sequence of each gene (Supplementary Tables 2 and 3). The protocol was programmed as follows: 95 °C for 10 min for 1 cycle; 95 °C for 15 s and 60 °C for 1 min for 40 cycles. Each sample was run in duplicate and the mean threshold cycle (Ct) was determined from the 2 runs. Gene expression was calculated from the difference of Ct between the target gene and endogenous housekeeping gene glyceraldehyde 3-phosphate dehydrogenase (GAPDH) as ΔCt. Subsequently, the ΔΔCt values were calculated by subtracting the mean ΔCt of the control group from those of the experimental groups, and the relative gene expression is expressed as the fold difference (2^-ΔΔCt^)^41,42^.

### Human neuronal cell cultures

The human neuroblastoma SH-SY5Y cell line was used for measurement of nerve fibre length as described^14,17^. The SH-SY5Y cells (ATCC#CRL-2266) was grown in Dulbecco’s modified Eagle’s medium/F12 (Life technologies Inc., Gaithersburg, MD, USA) supplemented with 10% FBS, 100 U/ml penicillin, and 0.1 mg/ml streptomycin^14,17,43^. Briefly, cells were seeded at a density of 2 × 10^3^ cells/ml in 12-well plates overnight, treated with 10 μM all-trans retinoic acid (RA) (Sigma) daily for 3 days, and then incubated with the following substances. Cells were incubated with bacteria-free intestinal supernatant and neurotrophins (NGF or BDNF, 100 ng/ml) in serum-free medium for 4 days, with fresh supernatant or neurotrophins being added every 2 days, for analysis of neurite outgrowth. Recombinant human β-NGF was purchased from R&D Systems (#256-GF), and human BDNF was purchased from Sigma-Aldrich (#B3795). In a separate experiment, RA-treated cells were incubated with 5-HT (1 μM; Sigma) or the 5-HT_7_ agonist LP211 (0.1 or 1 μM; Sigma) in culture medium containing reduced serum (2% FBS) for 2 days. Pharmacological inhibitors at various doses were added prior to stimulation with intestinal supernatant, 5-HT, or neurotrophins for analysis of neurite outgrowth.

For quantitative PCR analysis, SH-SY5Y cells were seeded at a density of 1 × 10^5^ cells/ml in 12-well plates overnight, treated with RA daily for 3 days and then incubated with 5-HT (1 μM) and LP211 (1 μM) in serum-reduced medium (2% FBS) for 24 or 48 h and with NGF (100 ng/ml) or BDNF (100 ng/ml) in serum-free medium for 24 h. Cellular RNA was extracted to assess gene transcript levels using primer pairs (Supplementary Table 2).

### Gene knockdown by transduction with lentiviral delivery of shRNA

The lentivirus containing short-hairpin RNA (shRNA) was assembled from shRNA oligonucleotides targeting *HTR7, NTRK1, or NTRK2* gene in a modified pLKO.1-puromycin vector, packaging plasmids (pCMV-R8.74psPAX2), and envelope plasmids (pMD2.G), which were constructed in the RNA Technology Platform and Gene Manipulation Core in Academia Sinica (Taipei, Taiwan)^36,38^. Briefly, bacterial broth with each lentiviral plasmid was streaked on agar plates, and single colonies were picked and incubated in terrific broth (BioShop, Burlington, ON, Canada) for plasmid extraction. For generating the lentiviral medium, HEK293T cells seeded for one day were transfected with the aforementioned three plasmids in an incubator for 24 h followed by replacement with culture medium for two days. All lentiviral medium produced from HEK293T cells was filtered through a 0.45-µm pore size filter and stored until use^36,38^.

For the shRNA-mediated gene knockdown experiment, SH-SY5Y cells seeded at 10^5^ cells/ml for 1 day were infected with the lentiviral medium mixed with culture medium at a ratio of 1:1 with 8 µg/mL polybrene (Sigma) for 2 days. The cells were then selected by culturing in medium containing 8 µg/mL puromycin (Sigma) for 48 h. The gene knockdown efficiency was confirmed by PCR and immunostaining.

### Preparation of bacteria-free mouse colonic supernatant

Colonic tissues (1 cm) were excised and homogenized in serum-free medium at a ratio of 1 mg of tissue to 10 µl of medium on ice as described^17^. One tablet of complete-Mini® (C-M) (Roche, Mannheim, Germany) was dissolved in 10 ml of serum-free medium for tissue homogenization. A protease inhibitor cocktail was used to prevent the proteolytic activity of the gut supernatant which may result in the cell death of cultured SH-SY5Y cells. Tissue lysates were centrifuged at 10,000 × *g* for 10 min at 4 °C, and the supernatants were carefully collected. The supernatants were mixed with a 20-fold volume of serum-free medium with C-M and passed through a sterilized filter with a pore size of 0.45 µm (Merck Millipore, Darmstadt, Germany) to remove bacteria. The bacteria-free supernatant was diluted with serum-free medium without C-M at a ratio of 1:100 and then added to RA-treated SH-SY5Y cells.

### Analysis of neurite outgrowth

Nerve fibre length was measured in SH-SY5Y cells following established methods^17^. Briefly, SH-SY5Y cells were incubated with mouse intestinal supernatant or other substances, and photographed using a light microscope equipped with a digital camera. The length of the nerve fibres was determined using imaging software (ImageJ 1.47 v). A total of 250–300 neurons per treatment group were analysed to obtain the average nerve fibre length and the percentage of neurons with fibres longer than 50 μm.

### Statistical analysis

All values were expressed as mean ± SEM. When more than three groups were compared, the one-way analysis of variance was chosen to examine differences between groups and Tukey multiple comparison test or Student-Newman-Keuls test was selected as a post-hoc test where applicable to determine the *P* value (Graph Pad Prism v. 5.01). Unpaired *t*-test with Welch’s test is adopted when the two group of samples are unpaired and are normally distributed. Significance was established at *P* < 0.05.

## Results

### Colonic expression of 5-HT_7_ on mucosal nerve fibres of IBS patients

**Fig. 1:**
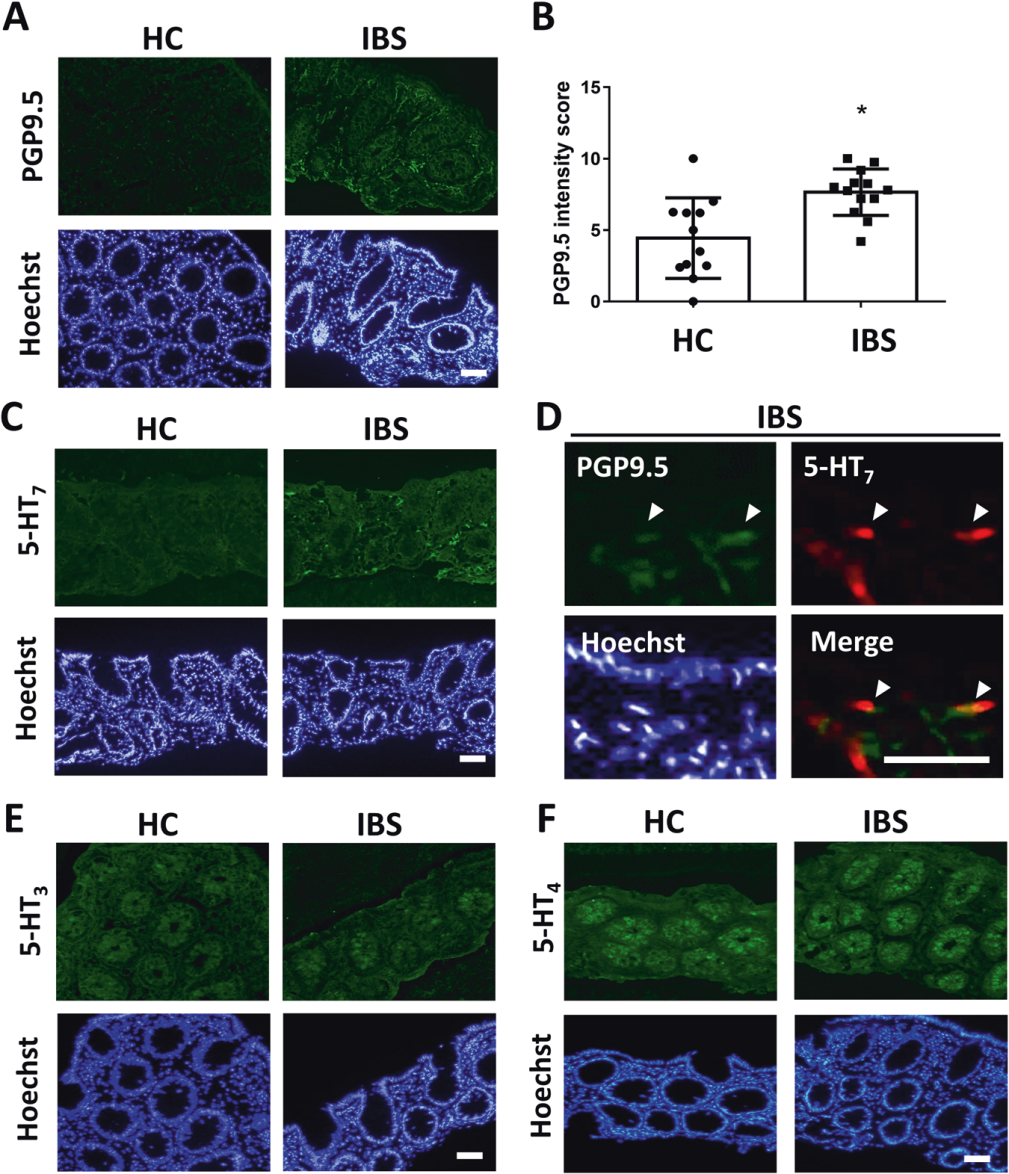
Immunostaining of nerve fibres in colonoscopic biopsy specimens from healthy subjects and IBS patients for PGP9.5 and 5-HT receptor subtypes. **A** Representative immunofluorescence images of staining for PGP9.5 (a pan neuronal marker, green) and nuclear staining with Hoechst dye (blue) in colonic mucosal biopsies from healthy control (HC) subjects (*N* = 12) and IBS patients (*N* = 13). Bar: 50 μm. **B** Quantification of PGP9.5 immunofluorescence intensity in mucosal area. Two to three images were taken for each individual, and an average of 30 images per group was scored in a blinded manner. **C** Staining for 5-HT_7_ (green) and with Hoechst dye (blue) in colonic biopsies. Bar: 50 μm. **D** Merged images of PGP9.5 (green), 5-HT_7_ (red) and Hoechst (blue) staining in colonic mucosal tissues from IBS patients. Arrowheads indicate the colocalization of PGP9.5 and 5-HT_7_. Bar: 10 μm. **E** Staining for 5-HT_3_ (green) and Hoechst (blue) in colonic biopsies. Bar: 50 μm. **F** Staining for 5-HT_4_ (green) and Hoechst (blue) in colonic biopsies. Bar: 50 μm.

Colonoscopic biopsy specimens were collected from IBS patients and healthy subjects (Supplementary Table 1), and stained for PGP9.5 (a pan-neuronal marker) and 5-HT receptor subtypes to assess their distribution in nerve fibres in the intestinal mucosa. Higher immunoreactivity to PGP9.5 in puncta and fibre-like patterns were observed in the lamina propria in biopsies from IBS patients than in those from healthy subjects (Fig. 1A and B). Punctate staining of 5-HT_7_ was localized to mucosal nerve fibres in IBS specimens, whereas low to negligible levels of 5-HT_7_ were noted in healthy mucosa (Fig. 1C and D). Immunostaining for 5-HT_3_ and 5-HT_4_ was mainly observed on colonic epithelia, and the expression patterns were comparable between IBS patients and healthy subjects (Fig. 1E and F).

### Activation of 5-HT_7_ is involved in intestinal hyperalgesia using two mouse models

**Fig. 2:**
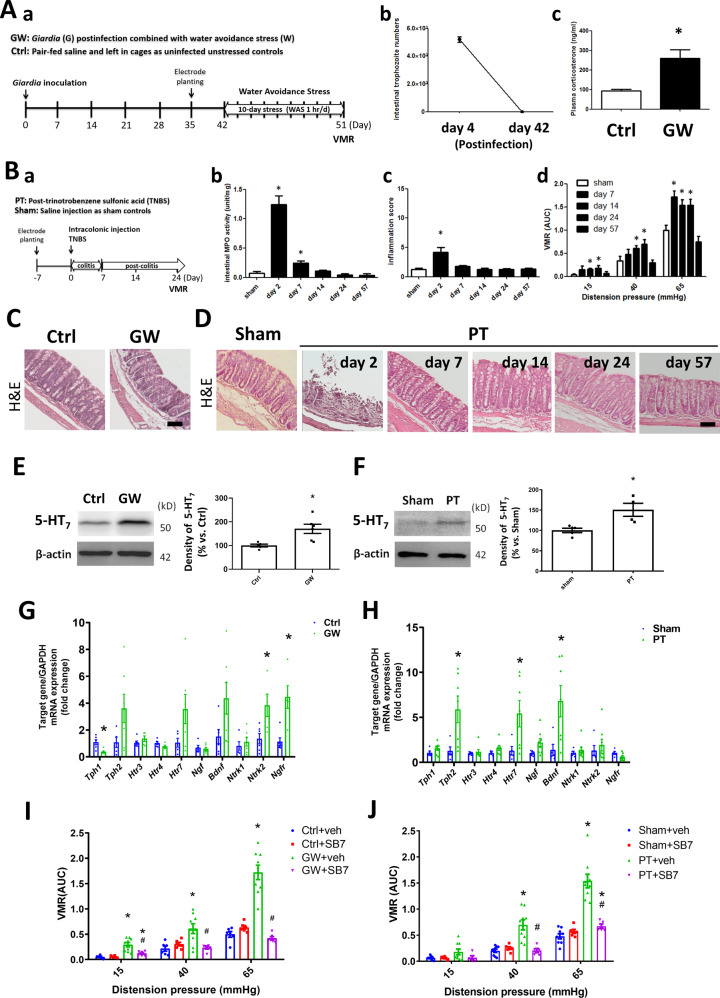
Activation of 5HT_7_ played a critical role in intestinal hyperalgesia in two IBS-like mouse models. Two mouse models with visceral hypersensitivity were investigated. **A** Timeline of the first model. **a** Mice were post-infected with *Giardia* and subjected to water avoidance stress (designated the GW model). The uninfected unstressed control (Ctrl) groups were pair-fed saline and left in cages unhandled. Electrode planting was performed at least 14 days prior to measurement of visceromotor responses (VMRs) to colorectal distension. **b** Pilot studies indicated the presence of *Giardia* trophozoites in the intestine on postinfection day 4 and clearance around 14 days. The absence of trophozoites in intestine were noted on postinfection day 42, confirming the self-limiting status of infection. **c** The plasma corticosterone levels as an indicator of psychological stress. *P < 0.05 *vs*.  Ctrl. *N* = 9–10/group. **B** Timeline of the second model. **a** Mice were intracolonically injected trinitrobenzene sulfonic acid (TNBS) and after resolution of colitis, the VMRs to colorectal distension was measured (designated the Post-TNBS (PT) model). The sham groups (Sham) were injected the same volume of saline. **b** and **c** Pilot studies showing myeloperoxidase (MPO) activity and histopathological score in mouse colonic tissues on various days after TNBS administration. **d** Pilot studies showing VMRs expressed as the area under curve (AUC) on various days after TNBS administration. Mice post-resolution of colitis on day 24 was chosen as the time point for the PT model. *P < 0.05 *vs*. Sham. *N* = 6–10/group. **C**, **D** Representative images of colon histology in Ctrl and GW mice, as well as those in Sham and PT mice after TNBS administration for 2, 7, 14, 24, and 51 days. Bar: 50 μm. **E**, **F** Western blots showing increased 5-HT_7_ protein levels in the colonic mucosal tissues of GW and PT mice compared to those of their respective controls. **G**, **H** Quantitative PCR results of transcript levels of tryptophan hydrolase 1/2 (*Tph1/2*), 5-HT receptor subtypes (*Htr3*, *Htr4*, and *Htr7*), nerve growth factor (*Ngf*), brain-derived neurotrophic factor (*Bdnf*), and receptor subunits of p75^NTR^ (*Ngfr*), TrkA (*Ntrk1*), and TrkB (*Ntrk2*) in the colonic tissues of GW and PT mice. *P < 0.05 vs. Ctrl or Sham. *N* = 6/group. **I**, **J** Intestinal pain levels in GW and PT mice after intraperitoneal (i.p.) treatment with a selective 5HT_7_R antagonist SB269970 (SB7) or vehicle (veh) 90 min prior to pain measurement. *P < 0.05 vs. Ctrl+veh or Sham+veh. ^#^*P* < 0.05 *vs*. GW+vehicle or PT+vehicle. *N* = 6–9/group.

The role of 5-HT_7_ in intestinal pain was evaluated in two animal models of IBS-like VH. The first model (designated the GW model) was established in our laboratory by subjecting mice to psychological stress of water avoidance in the post-clearance phase of giardiasis^17,30^ (Fig. 2A-a). Clearance of *Giardia* trophozoites in the intestine and perception of psychological stress were confirmed in the pilot study (Fig. 2A-b, A-c). Normal intestinal histology was observed in the GW model on the day measuring VMRs to colorectal distension (Fig. 2C). The second model (designated the PT model) was based on previous studies using postinflammatory mice that had recovered from TNBS-induced colitis^18,33^ (Fig. 2B-a). Persistent pain after resolution of colitis two weeks post-TNBS were confirmed in the pilot study (Fig. 2B-b, –c, and –d). The PT model on the day 24 exhibited normal intestinal histology without inflammation and was chosen for further studies of intestinal pain (Fig. 2D).

Western blotting showed higher expression of 5-HT_7_ proteins in colonic mucosal samples from the GW and PT mice than in those from their respective controls (Fig. 2E and F). The transcript levels of tryptophan hydroxylases (TPHs), 5-HT receptor (HTR) subtypes, neurotrophins, and neurotrophin receptor (NTR) subunits were also assessed in the whole gut tissues of GW and PT mice by quantitative PCR analysis. The TPHs are rate-limiting enzymes for serotonin synthesis, comprised of TPH1 which is mainly from non-neuronal sources such as enterochromaffin cells, and TPH2 which is from central and enteric neurons^8^. The NTR subunits include the high affinity receptors, i.e., Tropomyosin receptor kinase (Trk) A and TrkB, complex with the low affinity receptor p75^NTR^, for binding to NGF and BDNF, respectively^44^. Significantly higher *Ntrk2* and *Ngfr* gene expression were observed in the colonic tissues of GW mice than those of control mice (Fig. 2G), suggesting increased colonic TrkB and p75^NTR^ levels in the GW model. An increasing trend in *Tph2*, *Htr7*, and *Bdnf* transcripts but without statistical significance was shown in the colonic tissues of GW mice (Fig. 2G). Moreover, significantly higher *Tph2*, *Htr7*, and *Bdnf* gene expression were observed in the colonic tissues of PT mice compared to those of sham groups (Fig. 2H). As for genes encoding for other 5-HT receptor subtypes, no difference in *Htr3* or *Htr4* expression was seen in the colonic tissues of GW and PT mice compared to their respective control mice (Fig. 2G, H).

The increased VMRs of GW mice to colorectal distension was attenuated by i.p. administered SB-269970 (SB7, a selective 5-HT_7_ antagonist) (Fig. 2I). Administration of SB7 (i.p.) also significantly inhibited the VMRs of the PT mice (Fig. 2J). No difference in the baseline VMRs of their respective control groups was observed following i.p. administration of SB7 (Fig. 2I, J). The data suggested increased expression of TPH2 and 5-HT_7_ associated with higher levels of neurotrophin and NTR in the mouse colons. Moreover, 5-HT_7_ was involved in the visceral hypersensitivity of the IBS-like mouse models.

### Oral administration of a novel 5-HT_7_ receptor ligand attenuated intestinal pain

**Fig. 3:**
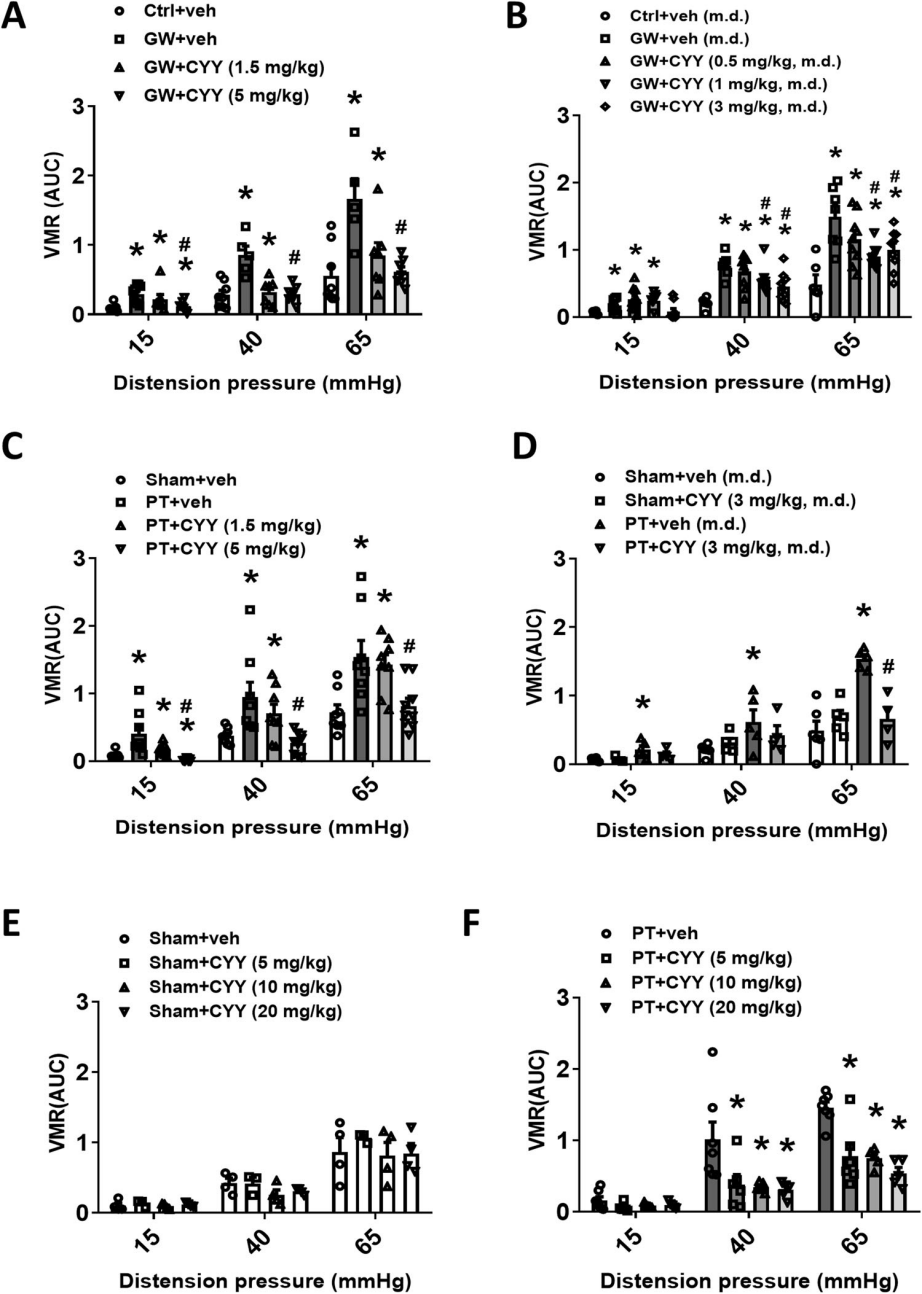
Oral administration of a novel 5-HT_7_ receptor ligand exerted potent analgesic effects in two IBS-like animal models. Mice were perorally (p.o.) administered CYY1005 (CYY) at various doses for the measurement of intestinal pain. **A** Analgesic effects of p.o. administered single dose of CYY (1.5 and 5 mg/kg) in the GW model. *P < 0.05 vs. Ctrl+vehicle, ^#^*P* < 0.05 vs. GW+vehicle. **B** Analgesic effects of p.o. administered multiple dose (m.d.) of CYY (1 and 3 mg/kg) for ten consecutive days in the GW model. *P < 0.05 vs. Ctrl+vehicle (m.d.), ^#^*P* < 0.05 vs. GW+vehicle (m.d.). **C** Analgesic effect of p.o. administered single dose of CYY (5 mg/kg) in the PT model. *P < 0.05 vs. Sham+vehicle, ^#^*P* < 0.05 vs. PT+vehicle. **D** Analgesic effects of p.o. administered multiple dose (m.d.) of CYY (3 mg/kg) for ten consecutive days in the PT model. No effects on the baseline values were seen in Sham control mice administered m.d. of CYY. *P < 0.05 vs. Sham+vehicle (m.d.), ^#^*P* < 0.05 vs. PT+vehicle (m.d.). **E** Administration of CYY at concentrations of 5, 10, or 20 mg/kg for single dose p.o. had no effect on the baseline values in Sham control mice. **F** Administration of CYY higher than 5 mg/kg for single dose p.o. did not result in more robust analgesic effects in PT mice. ^#^*P* < 0.05 *vs*. PT+vehicle. *N* = 6–8/group.

The analgesic effect of a newly synthesized 5-HT_7_ receptor ligand CYY1005 (CYY) was examined in the GW and PT models. First, a single dose of CYY was administered through an oral route at a concentration of 0, 1.5, or 5 mg/kg to the GW mice, and showed inhibition of VMRs in a dose-dependent manner (Fig. 3A). Administration of CYY (5 mg/kg, p.o.) decreased the VMRs of the GW mice to a level comparable to the control groups (Fig. 3A). We also tested multiple doses (m.d.) of CYY at a concentration of 0.5, 1, or 3 mg/kg for ten consecutive days. Peroral administration of CYY (1 and 3 mg/kg, m.d.) but not the lower concentration (0.5 mg/kg, m.d.) alleviated intestinal pain in GW mice (Fig. 3B).

In the second model, analgesic effects of single and multiple doses of CYY were evaluated. Peroral administration of a single dose of CYY at a concentration of 5 mg/kg inhibited the VMRs of PT mice, of which the pain levels were comparable to those of sham groups (Fig. 3C). Multiple doses of CYY (3 mg/kg) for ten consecutive days also decreased the VMRs of PT mice to a comparable level of the sham groups (Fig. 3D). In addition, CYY concentrations higher than 5 mg/kg were also assessed for their analgesic effects. Higher doses of p.o. administered CYY (10 and 20 mg/kg) neither cause a further decrease in the VMRs of PT mice nor altered the baseline VMRs of control groups (Fig. 3E and F).

### Neurite outgrowth to colonic mucosa was suppressed by multiple doses of CYY

**Fig. 4:**
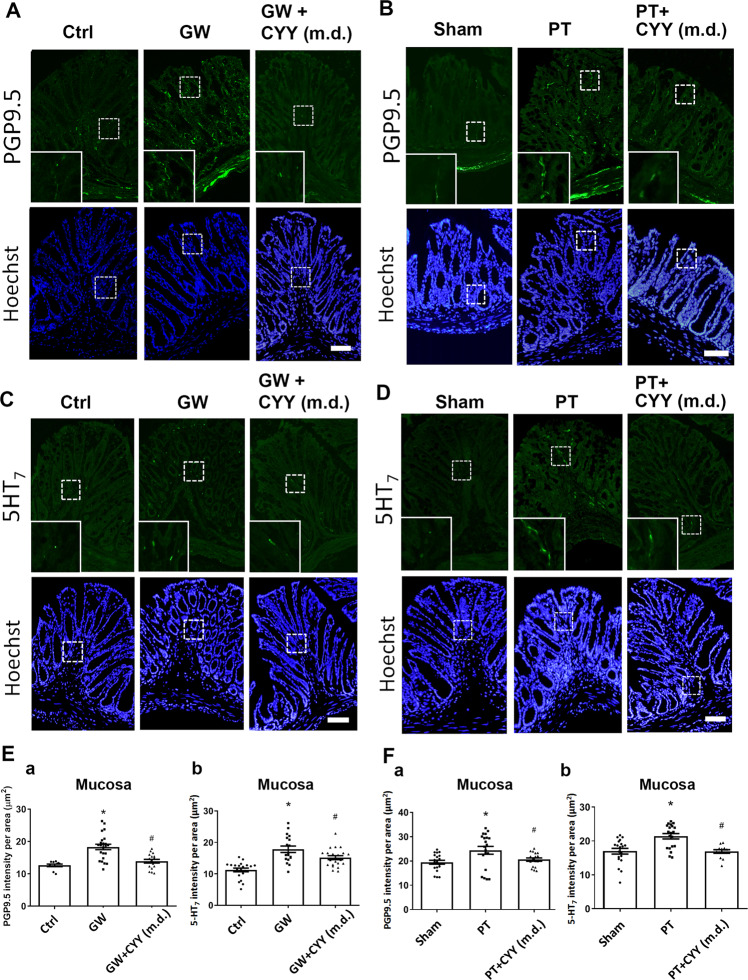
Increased mucosal nerve fibre densities were correlated with higher 5-HT_7_ expression in the colonic tissues of GW and PT mice. **A**,**B** Immunostaining for PGP9.5 in the colonic tissues of GW and PT mice and mice administered multiple doses (m.d.) of CYY. **C**, **D** Immunostaining for 5-HT_7_ in the colonic tissues of GW and PT mice and mice administered CYY (m.d.). The insets showed magnified images of the staining. Bar: 50 μm. **E**, **F** Quantification of immunoreactivity in the colonic mucosal tissues of GW and PT mice. Fluorescence intensity of (**a**) PGP9.5 and (**b**) 5-HT_7_ per area was quantified using an imaging software in a total of 25 images from each mouse group. *P < 0.05 vs. Ctrl or Sham; ^#^*P* < 0.05 vs. GW or PT. *N* = 6–8/group.

The mucosal nerve fibre density was visualized in the two mouse models using immunofluorescent staining. Consistent with the human data, increased PGP9.5 and 5-HT_7_ immunoreactivity was observed in the colonic mucosal tissues of GW and PT mice compared to respective control groups (Fig. 4). Furthermore, lower density of the mucosal nerve fibres with PGP9.5 and 5-HT_7_ immunoreactivity was noted in the GW and PT mice treated with multiple doses of CYY (3 mg/kg) for 10 consecutive days (Fig. 4), implicating that 5-HT_7_ was not only involved in neurotransmission for pain perception but also played a role in mucosal neurite outgrowth.

### Activation of 5-HT_7_ causes nerve fibre elongation in vitro

**Fig. 5:**
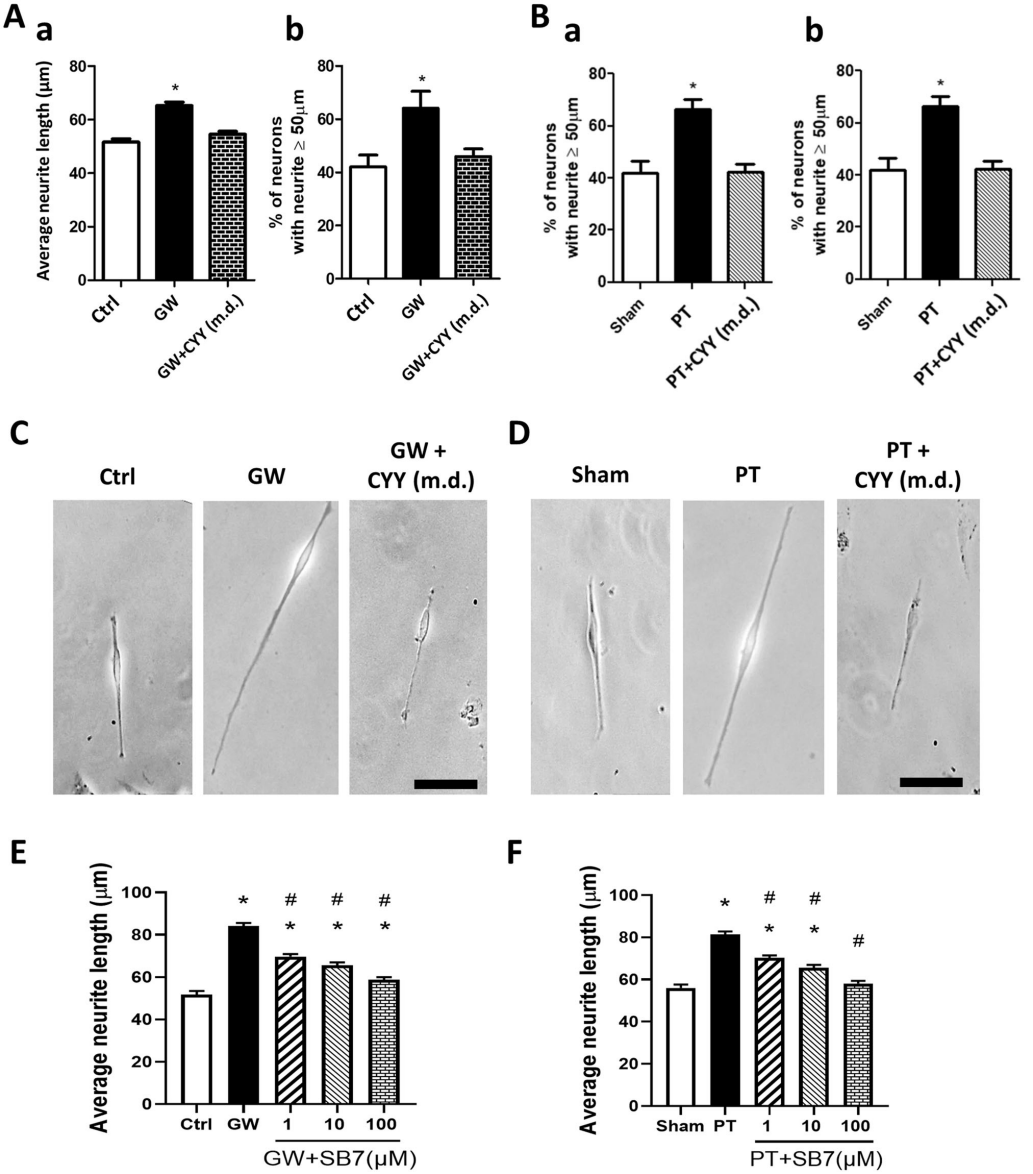
Mouse colonic supernatant induced neurite outgrowth in human SH-SY5Y cells in a 5-HT_7_-dependent manner. Differentiated SH-SY5Y cells were incubated with bacteria-free mouse colonic supernatant, and nerve fibre elongation was assessed. **A** Nerve fibre length of SH-SY5Y cells exposed to mouse colonic supernatant obtained from Ctrl, GW, or GW+CYY (m.d.) mice. **a** Average neurite length, and (b) Percentage of neurons with neurite longer than 50 μm. **B** Nerve fibre length of SH-SY5Y cells exposed to mouse colonic supernatant obtained from sham, PT, or PT+CYY (m.d.) mice. **a** Average neurite length, and **b** Percentage of neurons with neurite longer than 50 μm. **C**, **D** Representative figures of neurons after exposure to mouse colonic supernatant. Bar: 50 μm. **E**, **F** Pretreatment with a selective 5-HT_7_ antagonist SB7 inhibited neurite outgrowth caused by incubation with colonic supernatant from GW and PT mice in a dose-dependent manner. A total of 300–400 neurons from each group were used for quantification of nerve fibre length. The bar graph represented the mean ± SEM of each group. *P < 0.05 vs. Ctrl or Sham. ^#^*P* < 0.05 vs. GW or PT.

The molecular mechanisms of 5-HT_7_-dependent mucosal neurite outgrowth were investigated using a well-established human neuronal cell line. SH-SY5Y neuroblastoma cells differentiated by retinoic acid were incubated with bacteria-free mouse colonic supernatant (CS) to verify the action of gut-derived factors for promoting fibre extension. An increase in the average nerve fibre length was observed in neurons after incubation with CS obtained from GW mice (65.3 ± 1.3 μm) and PT mice (64.1 ± 1.3 μm) compared to CS obtained from control mice (51.6 ± 1.2 μm) and sham mice (53.3 ± 1.1 μm), respectively (Fig. 5A–D). The average nerve fibre length after incubation with control and sham CS was comparable to that of neurons differentiated by retinoic acid (53.4 ± 1.8 μm). Moreover, a higher percentage of neurons had fibres longer than 50 μm in the group incubated with CS from GW and PT mice than those incubated with CS from the control and sham mice, respectively (Fig. 5A and B). In contrast, neurons incubated with CS obtained from GW and PT mice that were treated with multiple doses of CYY (p.o.) did not exhibit longer nerve fibres (Fig. 5A–D).

A direct role of 5-HT_7_ in mouse CS-induced neurite outgrowth of SH-SY5Y cells was then verified by using a selective antagonist in vitro. Pretreatment with SB7 inhibited the nerve fibre elongation caused by CS from GW and PT mice in a dose-dependent manner (Fig. 5E and F). The results indicated that gut-derived factors in mouse CS activated 5-HT_7_ on SH-SY5Y cells for neurite elongation.

**Fig. 6:**
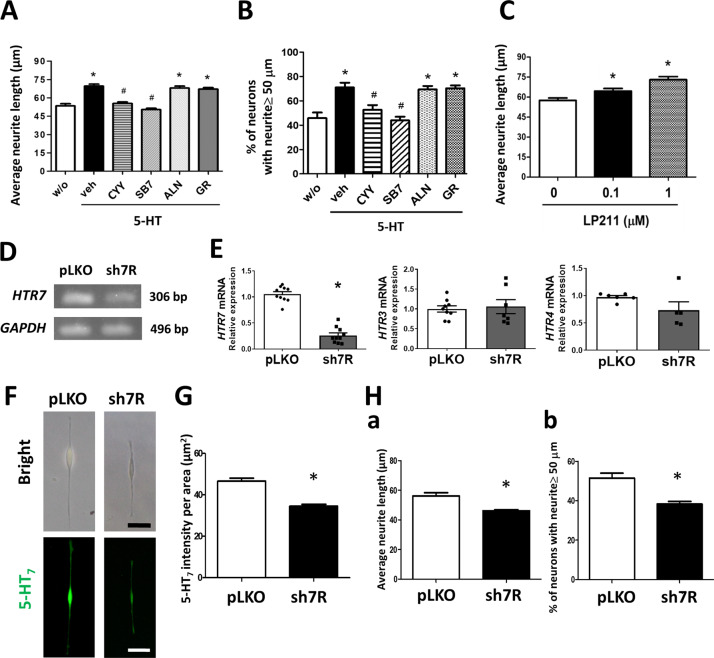
Stimulation with exogenous serotonin induced neurite outgrowth and neurotrophin upregulation in a 5-HT_7_-dependent manner. Human neuroblastoma SH-SY5Y cells differentiated by retinoic acid were stimulated with 5-HT (1 μM) for 48 h. **A**, **B** Average nerve fibre length and percentage of neurons with neurite longer than 50 μm in SH-SY5Y cells stimulated with 5-HT, in the absence or presence of CYY (10 μM, a 5-HT_7_ receptor ligand), SB7 (10 μM, a 5-HT_7_ antagonist), ALN (10 μM, a 5-HT_3_ antagonist), and GR (10 μM, a 5-HT_4_ antagonist). A total of 250–300 neurons from each group were used for quantification of nerve fibre length. The bar graph represented the mean ± SEM of each group. *P < 0.05 vs. w/o; ^#^*P* < 0.05 vs. 5-HT + vehicle. **C** Nerve fibre length after stimulation with a 5-HT_7_ agonist LP211 (0, 0.1, or 1 μM). *P < 0.05 vs. 0. **D** Gel images showing gene silencing of *HTR7* in SH-SY5Y cells by infection with lentiviral shRNA oligonucleotides (sh7R) compared to those with mock plasmid controls (pLKO). The bands are products of semi-quantitative PCR. **E** Quantitation PCR results indicated that only *HTR7*, but not *HTR3* or *HTR4*, gene expression was knocked down by sh7R. *P < 0.05 *vs*. pLKO. *N* = 6/group. **F** *P*hotoimages showing reduced 5-HT_7_ protein expression and nerve fibre length in the *HTR7*-knockdown cells. Bright field views (upper panel) and Immunostaining of 5-HT_7_ (lower panel). Bar: 50 μm. **G** Quantitative results of 5-HT_7_ intensity per area in the *HTR7*-knockdown cells. *P < 0.05 vs. pLKO. **H** Nerve fibre length in the *HTR7*-knockdown cells. **a** Average fibre length. **b** Percentage of neurons with neurite longer than 50 μm. A total of 250–300 neurons from each group were used for analysis of the gene knockdown experiments. The bar graph represented the mean ± SEM of each group. *P < 0.05 vs. pLKO.

To clarify whether serotonin per se exerted neurite outgrowth, exogenous 5-HT was added to the differentiated SH-SY5Y cells. Stimulation with 5-HT statistically increased the nerve fibre length (69.6 ± 1.6 μm) compared to those without stimulation (49.1 ± 2.5 μm) (Fig. 6A and B). The 5-HT-induced neurite outgrowth was inhibited by pretreatment with an antagonist or receptor ligand of 5-HT_7_, i.e., SB7 (50.3 ± 1.0 μm) and CYY (55.3 ± 1.4 μm) (Fig. 6A and B). In contrast, inhibitors of 5-HT_3_ (ALN) and 5-HT_4_ (GR) had no effect on the 5-HT-induced nerve fibre elongation (Fig. 6A, B). Administration of LP211 (a 5-HT_7_ agonist) also caused nerve fibre elongation (73.0 ± 2.3 μm) (Fig. 6C). Finally, gene silencing of *HTR7* by lentivirus-mediated shRNA transfection, as evidenced by lower expression of 5-HT_7_, decreased the length of nerve fibres (Fig. 6D-H).

### Enhancement of neurotrophin activity by serotonin binding to 5-HT_7_

**Fig. 7:**
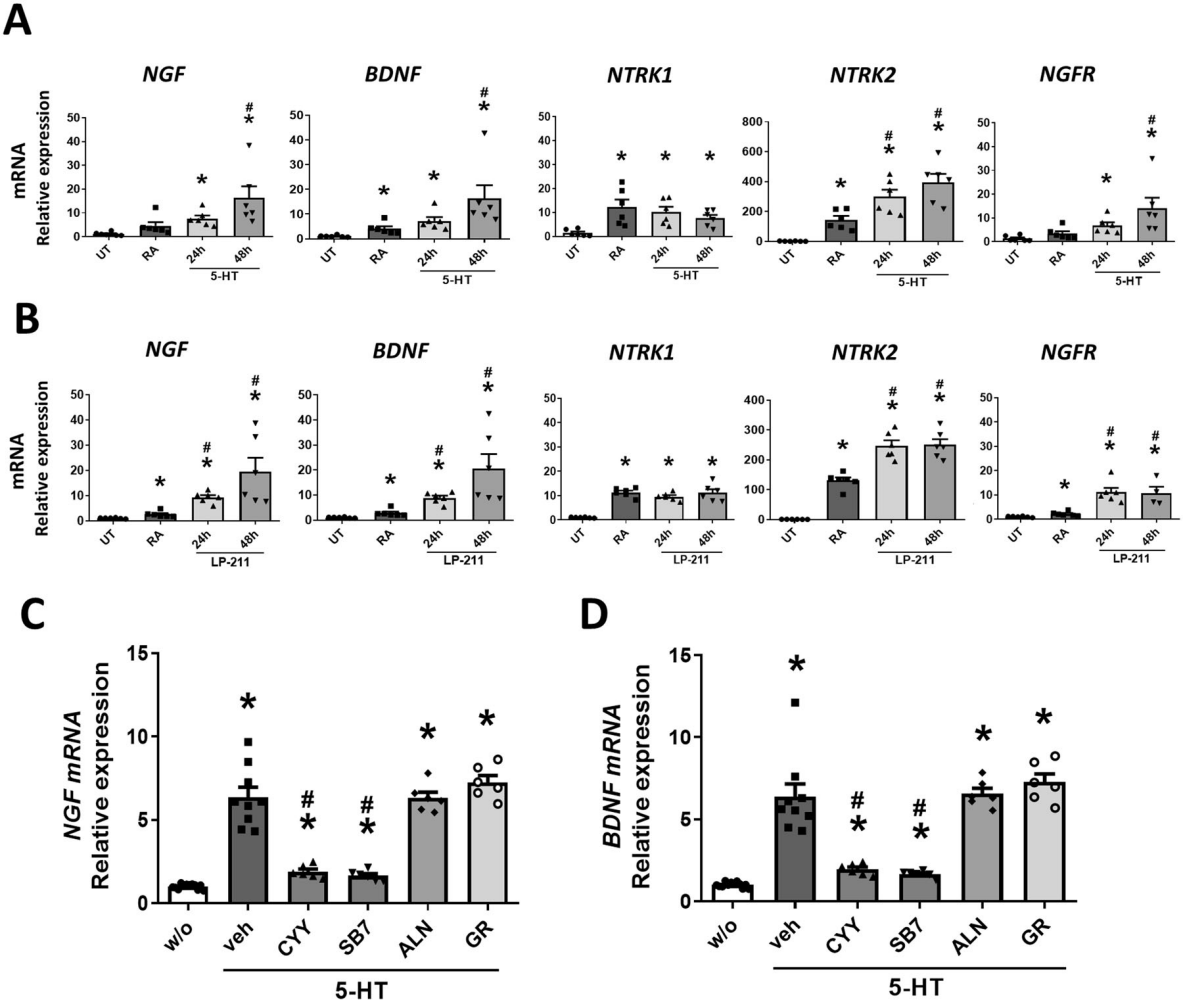
Enhanced neurotrophin expression by activation of 5-HT_7_. **A**, **B** Transcript levels of NGF and BDNF, and receptor subunits TrkA (*NTRK1*), TrkB (*NTRK2*), and p75^NTR^ (*NGFR*) in SH-SY5Y cells after exposure to 5-HT and LP211 for 24 and 48 h. The neuroblastoma cells which are untreated (UT) or those differentiated with retinoic acid (RA) served as controls. *P < 0.05 vs. UT; ^#^*P* < 0.05 vs. RA. *N* = 6/group. **C** The 5-HT-induced upregulation of *NGF* and *BDNF* gene expression was inhibited by 5-HT_7_ antagonists (CYY or SB7) but not by antagonists to 5-HT_3_ (ALN) or 5-HT_4_ (GR). *P < 0.05 *vs*. w/o; ^#^*P* < 0.05 vs. 5-HT + vehicle. *N* = 6/group.

Since serotonin was not a classical neurotrophic factor, the regulatory effect of 5-HT_7_ on neurotrophins and NTR subunit expression was assessed in SH-SY5Y cells. The addition of 5-HT and LP211 increased *NGF* and *BDNF*, as well as *NTRK2* and *NGFR* gene expression in neurons by quantitative PCR analysis (Fig. 7A and B). No change in *NTRK1* gene expression was seen by 5-HT or LP211 (Fig. 7A and B). Retinoic acid as a neuronal differentiation factor upregulated the transcript levels of all NTR subunits compared to those of undifferentiated cells (Fig. 7A and B). Next, the 5-HT receptor subtypes responsible for modulating neurotrophin transcript levels were examined. Pretreatment with 5-HT_7_ antagonists (CYY and SB7) blocked the 5-HT-mediated upregulation of *NGF* and *BDNF* gene expression, whereas inhibitors of 5-HT_3_ (ALN) and 5-HT_4_ (GR) had no effect on the neurotrophin levels (Fig. 7C and D). Taken together, the data suggested that activation of 5-HT_7_ upregulated the expression of neurotrophins and NTR subunits.

### Reciprocal aggravation between serotonin and neurotrophin pathways for neurite outgrowth

**Fig. 8:**
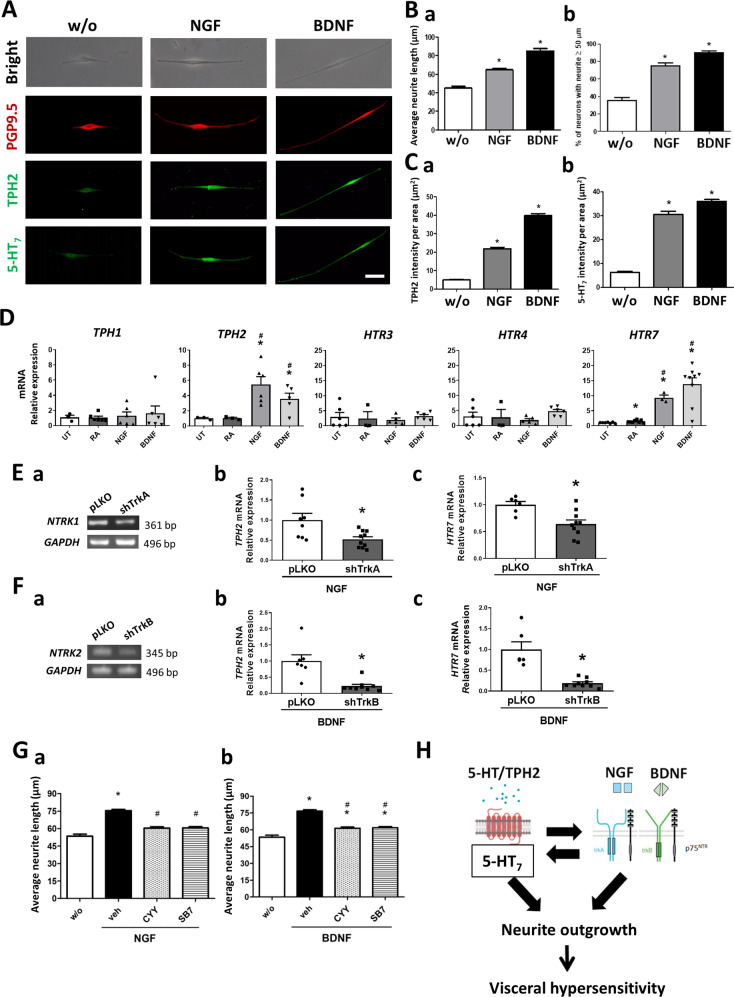
Reciprocal aggravation of serotonin and neurotrophin pathways via 5-HT_7_ activation in neurons. Human SH-SY5Y cells were stimulated with NGF or BDNF (100 ng/ml) for 48 h and the neurite length and protein levels were measured. **A** Representative immunostaining images of PGP9.5, TPH2, and 5-HT_7_ in cells with or without neurotrophin stimulation. Bar: 50 μm. **B** Longer nerve fibre length was observed after neurotrophin stimulation. **a** Average nerve fibre length and **b** percentage of neurons with neurite longer than 50 μm. *P < 0.05 *vs*. w/o. **C** Increased immunofluorescent intensity of (**a**) TPH2 and (**b**) 5-HT_7_ in neurons after neurotrophin stimulation. *P < 0.05 vs. w/o. A total of 250–300 neurons from each group were used for quantification. The bar graph represented the mean ± SEM of each group. **D** Transcript levels of tryptophan hydroxylases (*TPH1* and *TPH2*) and 5-HT receptor subtypes (*HTR3, HTR4*, and *HTR7*) in neurons after neurotrophin stimulation. The neuroblastoma cells which are untreated (UT) or those differentiated with retinoic acid (RA) served as controls. *P < 0.05 vs. UT; ^#^*P* < 0.05 *vs*. RA. *N* = 6/group. **E** Knockdown of *NTRK1* gene in SH-SY5Y cells by lentiviral shRNA targeting TrkA (shTrkA) suppressed the NGF-induced *TPH2* and *HTR7* overexpression. The mock plasmid control is labeled as pLKO. **a** Gel images of semi-quantitative PCR band products showing gene silencing of *NTRK1*. **b**, **c** Quantitative PCR results showing reduction of *TPH2* and *HTR7* mRNA levels in the *NTRK1*-knockdown cells upon NGF stimulation. **F** Knockdown of *NTRK2* gene by lentiviral shRNA targeting TrkB (shTrkB) decreased the BDNF-induced *TPH2* and *HTR7* overexpression. **a** Gel images of semi-quantitative PCR band products showing gene silencing of *NTRK2*. **b**, **c** Quantitative PCR results showing reduction of *TPH2* and *HTR7* mRNA levels in the *NTRK2*-knockdown cells upon BDNF stimulation. *P < 0.05 *vs*. pLKO. *N* = 6/group. **G** Neurotrophin-mediated nerve fibre elongation was inhibited by pretreatment with 5-HT_7_ antagonists CYY and SB7. *P < 0.05 vs. UT; ^#^*P* < 0.05 vs. vehicle. A total of 250–300 neurons from each group were used for quantification. The bar graph represented the mean ± SEM of each group. **H** Proposed schema of a positive-feedback loop between serotonin and neurotrophin pathways via 5-HT_7_ activation for induction of neurite outgrowth and visceral hypersensitivity.

Stimulation with recombinant NGF and BDNF induced nerve fibre elongation in SH-SY5Y cells (Fig. 8A and B). In addition to the longer nerve fibres, higher immunofluorescent intensity of 5-HT_7_ and TPH2 were also observed on the cells following neurotrophin stimulation (Fig. 8A and C). The results implicated that neurotrophin activation may increase the neuronal 5-HT_7_ expression and serotonin synthesis. To investigate whether elevated protein levels were a result of transcriptional upregulation, we quantified the mRNA levels of *TPH* and *HTR* genes in the neurotrophin-stimulated cells. Elevated *TPH2* and *HTR7* transcript levels were observed in the neurons after stimulation with NGF and BDNF, but no change was noted in the *TPH1*, *HTR3* or *HTR4* level of the neurotrophin-stimulated cells (Fig. 8D). Moreover, knockdown of *NTRK1* abolished the NGF-induced *TPH2* and *HTR7* transcriptional upregulation (Fig. 8E), whereas knockdown of *NTRK2* diminished the BDNF-induced *TPH2* and *HTR7* transcriptional upregulation (Fig. 8F). The data supported that neurotrophin binding to their putative NTRs upregulated serotonin synthesis and specifically, the expression of 5-HT receptor subtype 7.

Lastly, SH-SY5Y cells were pretreated with 5-HT_7_ antagonists (CYY or SB7) prior to stimulation with recombinant NGF and BDNF to determine the involvement of 5-HT_7_ in the neurotrophin/NTR-mediated fibre outgrowth. Pretreatment with CYY or SB7 attenuated the nerve fibre elongation caused by neurotrophins (Fig. 8G). Overall, these findings suggested a positive-feedback loop between serotonin and neurotrophin pathways for intensifying nerve fibre elongation and exacerbating intestinal pain (Fig. 8H).

## Discussion

IBS represents a substantial clinical problem; current treatments are ineffective for intestinal pain. Serotonergic nerves have long been implicated in the pathogenesis of intestinal hypernociception^10–12^, and increased mucosal nerve fibre density were recently observed in colonoscopic biopsies from IBS patients^13–16^. In the present study, overexpression of 5-HT_7_ was involved in VH through potentiation of neurite outgrowth to the gut mucosa in mouse models. Moreover, serotonin and neurotrophin both played roles in mucosal nerve fibre elongation via a 5-HT_7_-dependent mechanism. Inhibition of 5-HT_7_ activation led to potent analgesic effects in mice with VH, which offered a new strategy for IBS pain management.

A number of IBS-like animal models, each with weaknesses and strengths with respect to translational value, have been established^17,18,30–33^. We demonstrated by using two IBS-like mouse models that peroral administration of a novel 5-HT_7_ antagonist CYY reduced intestinal hyperalgesia and attenuated the mucosal neurite outgrowth. Increased 5-HT_7_-expressing mucosal nerve endings associated with higher TPH2 gene expression (a neuronal enzyme for serotonin synthesis) were observed in the colon of GW and PT mice, of which neurite outgrowth was reversed by multiple doses of CYY. Here, we identified that 5-HT (beyond its classical role as a neurotransmitter) enhanced mucosal innervation and promoted intestinal hyperalgesia via activation of 5-HT_7_. Previous studies have shown that 5-HT_7_ was involved in synapse formation for cerebral neurons^45,46^. Others documented a role of 5-HT_7_ in regulation of sleep patterns, mood, and thermal responses in the brain using 5-HT_7_-knockout mice^47^. A recent study using mice deficient in TPH1 (a serotonin synthesis enzyme in nonneural cells) demonstrated that mucosal 5-HT was crucial for maintaining the integrity of myenteric neurons during early microbiota colonization period^48^. Therefore, the 5-HT_7_dependent increase of mucosal afferents likely originated from the enteric nervous system. Despite the clear indication of enhanced mucosal innervation in correlation with increased pain perception, the origins of mucosal afferents derived from either submucosal and myenteric nerve plexuses or dorsal root ganglia warrant further investigations.

Immunostaining in human biopsy specimens revealed that 5-HT_7_ was highly expressed in mucosal nerve fibres in IBS tissues while other receptor subtypes (i.e., 5-HT_3_ and 5-HT_4_) were found mainly on colonic epithelial cells. The cells types in human specimens stained with 5-HT_3_ and 5-HT_4_ such as surface epithelial or enteroendocrine cells required further investigation. Dense distribution of puncta- or fibre-like PGP9.5 staining was observed in the IBS biopsy specimens, suggesting mucosal neurite outgrowth in patients. Colocalization of PGP9.5 and 5-HT_7_ staining was noted in the IBS specimens and mouse colonic tissues. However, the distribution of 5-HT_7_ staining in IBS specimens was patchy and therefore, unable to be quantified. This could be due to scarcity of human samples or because that not all nerve fibres expressed 5-HT_7_. Amongst the subtypes, 5-HT_3_ antagonists and 5-HT_4_ agonists are used for the treatment of diarrhoeal-predominant and constipation-predominant IBS, respectively. 5-HT_3_ receptor is a ligand-gated ion channel, whereas 5-HT_4_ and 5-HT_7_ are G protein-coupled receptors. The roles of 5-HT_3_ and 5-HT_4_ in restoring defecation patterns in IBS patients have been extensively studied, and multiple lines of evidence indicate that these subtypes act via mucosal sites. The 5-HT_2A_, 5-HT_3_ and 5-HT_4_ had been localized to the gut epithelium in humans and mice^49–51^, and were involved in serotonin-mediated crypt proliferation and epithelial cell turnover^52–54^. It suggests that symptom alleviation by targeting these receptor subtypes is attributed to ion secretion, which is a main factor for determining liquid content and consistency of stool and hence controlling bowel movements. In other words, pharmacologically targeting 5-HT_7_-dependent mucosal neurite outgrowth represents a novel mode-of-action for managing intestinal pain.

Neurotrophins, which are mainly produced from neurons and glial cells, are crucial for neuron survival, repair, and nerve fibre elongation. Consistent with the transcript levels in IBS-like mouse colons, activation of 5-HT_7_ led to increased expression of NGF, BDNF, and NTR subunits TrkB and p75^NTR^ associated with longer nerve fibres in the human neural cell lines. Conversely, neurotrophin stimulation also elevated the levels of TPH2 and 5-HT_7_ without changing the 5-HT_3_ and 5-HT_4_ levels in neurons. This reciprocal upregulation of serotonin and neurotrophin systems via 5-HT_7_ activation was essential for nerve fibre elongation. It is noteworthy that blockade of 5-HT_3_ or 5-HT_4_ had no effect on neurotrophin-mediated neurite outgrowth, suggesting that this positive-feedback loop was specific to the 5-HT_7_ subtype. Although the presence of *HTR3* and *HTR4* gene transcripts were also found in neuronal SH-SY5Y cells, antagonists to 5-HT_3_ or 5-HT_4_ did not attenuate the serotonin- or neurotrophin-induced neurite outgrowth in our cell culture model. Previous studies demonstrated that activation of 5-HT_4_ by agonists applied locally at anastomotic sites of guinea pig ileum caused increases in neuronal numbers and fibre length in the enteric nervous system associated with more stem-like cells and 5-HT_4_- and bromodeoxyuridine-positive cells^55^, whereas other reports showed that 5-HT_4_ was involved in neurite retraction in neuroblastoma cells^56,57^. The discrepancy of results may be due to the use of different model systems such as neuronal cell lines containing a single cell type or whole gut tissues that have multiple cell types. Long-term use of the novel 5-HT_7_ antagonist CYY not only suppressed the abnormal mucosal nerve hypersensitivity but also inhibited 5-HT_7_ overexpression in the gut through curbing the feedback loop, suggesting a reduced risk of drug tolerance or insensitivity. To our knowledge, this is the first report to document a previously unknown intestinal neural circuit modulated by neurotrophins and serotonin, of which the two pathways merged at 5-HT_7_. The 5-HT_7_ could be an alternative target for reducing mucosal neurite outgrowth without affecting basal neurotrophic functions.

An aggravating loop between serotonin and neurotrophins was identified in this study for intensifying mucosal innervation and intestinal nociception. In addition to neurons, crosstalks between serotonin and neurotrophin were reported in epithelial cell types in a rodent model of neonatal maternal deprivation. This elegant study documented that early life stress causes overactivation of NGF/TrkA signalling and increases the differentiation of secretory progenitor cells, i.e. 5-HT-producing enterochromaffin cells^58^. It is noteworthy that a direct effect of NGF on epithelial cells was demonstrated by using three-dimensional primary organoid cultures^58^, of which the organoids were derived from colonic crypts and devoid of neurons. Recent studies demonstrated microbiota dybiosis associated with distinct neuroimmune signatures including upregulated 5-HT in colon biopsy of patients with functional abdominal pain^59,60^. Early studies reported enterochromaffin cell hyperplasia in post-infectious IBS patients following *Campylobacter enteritis* infection and also after *Trichinella spiralis* infection in mouse models^61,62^. The possibility of enterochromaffin cells as a source of serotonin cannot be ruled out in the crosstalks between serotonin and neurotrophins for neurite outgrowth. Whether serotonin derived from enterochromaffin cells may contribute to the upregulation of neurotrophin pathways in addition to neuronal sources warrant further investigation. Taken together, our findings and others supported a bidirectional aggravation of serotonin and neurotrophin pathways in multiple cell types, which may lead to chronic VH in the gut.

In summary, we conclude that mucosal neurite outgrowth contributed to intestinal hypernociception in IBS mouse models. A positive-feedback loop driving nerve fibre elongation was observed between serotonin and neurotrophins, whereby 5-HT_7_ plays a key role in the mechanism. More importantly, 5-HT_3_ and 5-HT_4_ were not involved in this new mode of action. Oral administration of a novel 5-HT_7_ antagonist attenuated pain sensation and mucosal innervation, which may be a promising therapeutic option for IBS-related pain.

## Supplementary information

Supplementary Tables

## Data Availability

The data and material are available upon request.
