# Supplementary material for: 5-HT7 receptor-dependent intestinal neurite outgrowth contributes to visceral hypersensitivity in irritable bowel syndrome
Source: Lab Invest. 2022 May 18;102(9):1023–37. doi: 10.1038/s41374-022-00800-z (PMC9420680; doi:10.1038/s41374-022-00800-z)
Supplement: Supplementary file 1 — Supplementary Tables [file 41374_2022_800_MOESM1_ESM.docx]

**Supplementary Tables**

**Suppl Table 1 Characteristics of healthy controls (HC) and diarrhoea-predominant IBS patients for colonoscopic biopsy collection.**

|  | **HC** | **IBS** | ***P*-values** |
| --- | --- | --- | --- |
| **Number of patients** | 12 | 13 |  |
| **Age, years (mean)** | 33-66 (46 ± 3.4) | 31-76 (56 ± 16.2) | 0.09 |
| **Gender, male; female** | 5;7 | 9;2 |  |
| **Weight, kilograms (mean)** | 50-84.0  (64.3 ± 13.9) | 48-76.0  (65.3 ± 8.5) | 0.83 |
| **Body mass index (kg/m^2^)** | 23.6 ± 3.9 | 22.9 ± 2.2 | 0.62 |
| **Days with loose or watery stool* (%)** | ― | 70.3 ± 30 |  |
| **Bowel frequency (daily, mean)** | ― | 4 ± 2.5 |  |
| **C-reactive protein, mg/L (median, range)** | ― | 0.3, 0.2-1.6 |  |

**Footnote:** *Stool consistency is classified as type 6 or 7 by using Bristol stool scale, based on patient recall over 7 to 30 days.

**Suppl Table 2 Primer pairs of quantitative PCR for mouse cells**

| **Protein** | **Gene** | **Oligonucleotide sequence (5’-3’)** | **Size (bp)** |
| --- | --- | --- | --- |
| Tph1 | *Tph1* | fp: TGCGACATCAGCCGAGAAC  rp: GGTGGCGTGCGACTTCA | 60 |
| Tph2 | *Tph2* | fp: CTCCCCCTGCTGACCAAGTA  rp: TCCAGTTGCGGCACGTT | 56 |
| 5-HT_3_ | *Htr3* | fp: ACCCAGGCCCGAGATACC  rp: AGTTAGCCAGGAGGTGGTCTGA | 64 |
| 5-HT_4_ | *Htr4* | fp: CACACATCGCATGAGGACAGA  rp: GCAGCCCATGATGACACATAA | 61 |
| 5-HT_7_ | *Htr7* | fp: TGCTGGCTGCCGTTTTTC  rp: CTACAGGAGGTGCCACAGATAAAG | 62 |
| Ngf | *Ngf* | fp: TTCCAGGCCCATGGTACAA  rp: GGTGGATGAGCGCTTGCT | 60 |
| Bdnf | *Bdnf* | fp: GGCCCAACGAAGAAAACCAT  rp: AGCATCACCCGGGAAGTG | 55 |
| TrkA | *Ntrk1* | fp: TCTGGGAGATCTTCACCTATGGA  rp: CGATCGCCTCAGTGTTGGA | 63 |
| TrkB | *Ntrk2* | fp: ACAGATCTCCGCTCACTTCATG  rp: GGGTAATTTGGGTTTGTCTCGTAGT | 63 |
| p75^NTR^ | *Ngfr* | fp: TCTTGGCTGCTGTGGTTGTG  rp: TGCAGCTGTTCCATCTCTTGA | 63 |
| Gapdh | *Gapdh* | fp: CATGGCCTTCCGTGTTCCTA  rp: GCGGCACGTCAGATCCA | 54 |

**Footnote:** All real-time PCR primers were designed in this study. The annealing temperature (*Tm*) for all primer pairs is set to 60°C.

**Abbreviations:** Tph, Tryptophan hydroxylase; 5-HT, 5-hydroxytryptamine; 5-HT_3/4/7_, 5-hydroxytryptamine receptor subtype 3/4/7; Ngf, Nerve growth factor; Bdnf, Brain-derived neurotrophic factor; Trk, Tropomyosin receptor kinase; Gapdh, Glyceraldehyde 3-phosphate dehydrogenase.

**Suppl Table 3 Primer pairs of quantitative PCR for human cells**

| **Protein** | **Gene** | **Oligonucleotide sequence (5’-3’)** | **Size (bp)** |
| --- | --- | --- | --- |
| TPH1 | *TPH1* | fp: GCTTCAGAGGAGGCTGTTCAA  rp: TTACATAGACCAAACTCCACAGTGAAA | 68 |
| TPH2 | *TPH2* | fp: TGGCAAAAATGACGACAAAGG  rp: CCACTTTCGGTAGCAGCTTCA | 63 |
| 5-HT_3_ | *HTR3* | fp: CTGCAGGAGCTGTCCTCCAT  rp: GGCCACCTCTCGGATCTCA | 63 |
| 5-HT_4_ | *HTR4* | fp: GTCGGCAGACCAGCATAGC  rp: AGGGTCTTGGCTGCTTTGG | 60 |
| 5-HT_7_ | *HTR7* | fp: GATCACAAGGCCCCTCACAT  rp: TCATCTTCGCCATGCATTTC | 59 |
| NGF | *NGF* | fp: GGGCGAATTCTCGGTGTGT  rp: GTCTGTGGCGGTGGTCTTATC | 64 |
| BDNF | *BDNF* | fp: CAAAAATTACCTAGACGCTGCAAA  rp: GCGGGCAGGGTCAGAGT | 64 |
| TrkA | *NTRK1* | fp: TTTCGTGGCGCCAGATG  rp: GAGACTCCAGAGCGTTGAAGGA | 77 |
| TrkB | *NTRK2* | fp: GGACACCACGAACAGAAGTAATGA  rp: TTCCCGACCGGTTTTATCAG | 64 |
| p75^NTR^ | *NGFR* | fp: CGAGGCACCACCGACAAC  rp: ACAACCACAGCAGCCAGGAT | 59 |
| GAPDH | *GAPDH* | fp: AACGGGAAGCTTGTCATCAATGGAA  rp: GCATCAGCAGAGGGGGCAGAG | 194 |

**Footnote:** All real-time PCR primers were designed in this study. The annealing temperature (*Tm*) for all primer pairs is set to 60°C.

**Abbreviations:** TPH, Tryptophan hydroxylase; 5-HT, 5-hydroxytryptamine; 5-HT_3/4/7_, 5-hydroxytryptamine receptor subtype 3/4/7; NGF, Nerve growth factor; BDNF, Brain-derived neurotrophic factor; Trk, Tropomyosin receptor kinase; GAPDH, Glyceraldehyde 3-phosphate dehydrogenase.
